# Distinct leaf transcriptomic response of water deficient *Eucalyptus grandis* submitted to potassium and sodium fertilization

**DOI:** 10.1371/journal.pone.0218528

**Published:** 2019-06-20

**Authors:** Bénédicte Favreau, Marie Denis, Raphael Ployet, Fabien Mounet, Hana Peireira da Silva, Livia Franceschini, Jean-Paul Laclau, Carlos Labate, Helaine Carrer

**Affiliations:** 1 CIRAD, UMR AGAP, Montpellier, France; 2 AGAP, Université de Montpellier, CIRAD, INRA, Montpellier SupAgro, Montpellier, France; 3 Laboratoire de Recherche en Sciences Végétales, Université de Toulouse, CNRS, UPS, Castanet-Tolosan, France; 4 Department of Genetics, Luiz de Queiroz College of Agriculture, University of São Paulo, São Paulo, Brazil; 5 CIRAD, UMR Eco&Sols, Montpellier, France; 6 Department of Biological Sciences, Luiz de Queiroz College of Agriculture, University of São Paulo, São Paulo, Brazil; ICAR-Indian Institute of Agricultural Biotechnology, INDIA

## Abstract

While potassium fertilization increases growth yield in Brazilian eucalyptus plantations, it could also increase water requirements, making trees more vulnerable to drought. Sodium fertilization, which has been shown to promote eucalyptus growth compared to K-deficient trees, could partially mitigate this adverse effect of potassium. However, little is known about the influence of K and Na fertilization on the tree metabolic response to water deficit. The aim of the present study was thus to analyze the transcriptome of leaves sampled from *Eucalyptus grandis* trees subjected to 37% rainfall reduction, and fertilized with potassium (K), sodium (Na), compared to control trees (C). The multifactorial experiment was set up in a field with a throughfall exclusion system. Transcriptomic analysis was performed on leaves from two-year-old trees, and data analyzed using multifactorial statistical analysis and weighted gene co-expression network analysis (WGCNA). Significant sets of genes were seen to respond to rainfall reduction, in interaction with K or Na fertilization, or to fertilization only (regardless of the water supply regime). The genes were involved in stress signaling, primary and secondary metabolism, secondary cell wall formation and photosynthetic activity. Our focus on key genes related to cation transporters and aquaporins highlighted specific regulation of ion homeostasis, and plant adjustment to water deficit. While water availability significantly affects the transcriptomic response of eucalyptus species, this study points out that the transcriptomic response is highly dependent on the fertilization regime. Our study is based on the first large-scale field trial in a tropical region, specifically designed to study the interaction between water availability and nutrition in eucalyptus. To our knowledge, this is the first global transcriptomic analysis to compare the influence of K and Na fertilization on tree adaptive traits in water deficit conditions.

## Introduction

Subtropical and tropical hardwood plantations are dominated by the genus *Eucalyptus*, which provides the raw material for wood, paper, charcoal, and biofuel products, as well as large quantities of firewood. While eucalyptus trees have been planted in a wide range of environmental conditions, their growth is highly dependent on soil fertility and water supply [1, 2]. In Brazil, large-scale commercial plantations have been a feature of the landscape since the early 1960s, and trees were selected for their growth and disease resistance in local conditions [3]. Most tropical eucalyptus plantations are established in nutrient-poor soils [4]. Like in many tropical countries, Brazilian forest plantations are already subject to climate change, with increasing variation in the frequency and intensity of rainfall [5]. The management of the plantations must thus be adapted to cope with biotic and abiotic stresses that are both predicted to increase dramatically [6, 7]. To maintain the productivity of eucalyptus plantations in southern Brazil, large amounts of potassium (K) fertilizers are generally applied to increase growth, yield and improve drought resistance [8, 9]. K is an essential monocation that specifically activates at least 60 enzymes involved in plant growth, transcription and protein translation, amino acid synthesis, and carbohydrate metabolism [10, 11]. K improves plant drought tolerance by regulating guard cells and turgor in motor cells, thereby helping the plant to better adjust its osmotic potential [12–14], mitigating photosynthesis inhibition through its positive impact on photo-oxidative stress [15, 16], and by enhancing plant carbon metabolism through increased sucrose content and sucrose partitioning [17–21]. These processes require maintenance of K homeostasis, with tight control of K uptake and efflux, involving a large number of selective and non-selective channels and transporters [22].

However, potassium (K) application has also been shown to increase tree water requirements due to improved tree growth [23]. To mitigate this adverse effect, K fertilizers could be replaced by a cheaper mix of sodium (Na) and K [4, 24], which has the additional advantage of being more accessible to small producers in poor tropical regions. While Na is toxic at high concentrations [25], low concentrations have been shown to promote growth in a number of salt tolerant species including wheat [26, 27], sugar beet [28, 29], and red beet [30, 31]. In *Eucalyptus grandis*, Na and K fertilization was shown to increase above-ground biomass, respectively, 1.5 and 2 times compared to K-deficient trees, at harvest (6 years after planting) [4, 32]. Na can replace K in biophysical properties and non-specific functions that take place in vacuoles, particularly at low K concentrations [33], where it participates in osmotic adjustment [34–36], stomatal conductance and photosynthesis [37, 38]. However, major metabolic processes take place in cytoplasm, requiring highly controlled K homeostasis (protein synthesis, photosynthesis, glycolysis) that cannot be fulfilled by Na [39]. The beneficial role of Na fertilization suggests that essential metabolic functions could be maintained, and/or finely regulated, but the underlying mechanisms remain to be deciphered [40, 41]. Few studies have evaluated the response of perennial plants when K is replaced by Na. In cacao trees, Na had a positive impact on assimilation rate and water use efficiency (WUE) [42]. In drought conditions, supplying small quantities of Na can have a positive impact on plant growth, especially when access to other nutrients, such as potassium, azote and phosphorus, is limited [43], as shown in olive [37] and eucalyptus trees [23, 44]. Therefore, comparing tree response to K and Na at the physiological and molecular level, in the context of water scarcity, is a step forward.

We performed a global transcriptomic analysis of leaves from 2-year-old *Eucalyptus grandis* trees, submitted to water deficiency combined with K or Na supply. The data set was first analyzed using multifactorial statistical approaches, after which significant genes were selected using standard pairwise comparison and weighted gene co-expression network analysis (WGCNA). WGCNA is a powerful tool to gain new insights into both the function of genes and the mechanisms that control complex traits [45, 46]. Co-expression gene analysis is based on the ‘guilt-by-association’ (GBA) principle, which states that genes with similar functional properties tend to interact and have similar expression profiles [47]. This methodology has been successfully used to study ripening in grape berry and citrus [48, 49] as well as drought tolerance in grapevine and rice [50, 51]. The statistical and bioinformatic approaches outlined in this paper were necessary to cope with the complexity of the data analysis and the interpretation of multifactorial transcriptomic data. The combined effect of water deficiency and fertilization was successfully broken down, and contrasted molecular processes were revealed. As the responses to water availability and mineral nutrition are highly regulated by ion transporters, we searched for K transporters and channels, and aquaporins, as they are known to be involved in K homeostasis and are important mediators of stress responses [52, 53]. Their involvement in the regulation of K and Na transport, in the context of water scarcity, is still under investigation [54]. Overall, global transcriptomic analysis highlighted key mechanisms involved in the tree’s response to nutrition and water availability and provides a new framework for further investigation.

## Material and methods

### Study site and experimental design

A split-plot experiment was set up in southern Brazil, at the Itatinga research station of the University of Sao Paulo. The experimental design is described in detail in Battie-Laclau [23]. Briefly, the soils are very deep Ferralsols (> 15 m) with a clay content ranging from 14% in the A1 horizon to 23% in deep soil layers [55]. Mean annual rainfall is 1,400 mm and mean temperature is 20°C. The climate is characterized by a rainy season lasting from October to May and a dry season from June to September (S1 Fig). Cuttings of a highly productive *E*. *grandis* clone (Suzano Company) were planted in June 2010 in three blocks covering a total of 2.5 ha. The whole plot factor was the rainfall regime: full rainfall (FR) vs reduced rainfall (RR). Rainfall was reduced by 37% by using a throughfall exclusion system made of plastic sheets. The split-plot factor was the fertilization regime, comparing a control treatment without K or Na addition (C), potassium addition (K) and sodium addition (Na). The individual subplots were 864 m^2^ in size, with 144 trees planted in one block at a spacing of 2 x 3 m for a specific water and fertilization regime (total of 432 trees per treatment). The total amounts of KCl and NaCl were applied three months after planting. All the trees in the experiment were fertilized with the other nutrients at planting (12 g N m-2, 3.3 g P m-2, 200 g m-2 of dolomitic lime and trace elements), which was non-limiting for tree growth at this site [56]. Six treatments were applied as follows:

C and RR, control nutrition, without K and Na application, and 37% of throughfall excluded;
Na and RR, 0.45 mol Na m^-2^ applied as NaCl, and 37% of throughfall excluded;
K and RR, 0.45 mol K m^-2^ applied as KCl, non-limiting in terms of the availability of K for tree growth (Almeida et al., 2010), and 37% of throughfall excluded;
C and FR, control nutrition, without K and Na application, and no throughfall exclusion;
Na and FR, 0.45 mol Na m^-2^ applied as NaCl, and no throughfall exclusion;
K and FR, 0.45 mol K m^-2^ applied as KCl and, no throughfall exclusion.

### Leaf sampling

Leaves were collected from two-year-old trees at the end of the rainy season. For each treatment in block 1, two-month-old fully expanded leaves were collected at the top of the crown between 8 and 11 am. Leaves from four biological replicates (4 trees) per treatment were sampled, and immediately frozen in liquid nitrogen, and conserved at -80°C until further analysis.

### Ecophysiological measurements

Soil water contents were measured weekly using three TDR probes (Trase Soilmoisture, Santa Barbara, CA, USA) installed at different distances from the trees to depths of 0.15, 0.50, 1.50, 3.00, 4.50 and 6.00 m in each subplot in block 1 [23]. Three piezometers at our study site showed that the depth of the water table was about 16 m deep in the period the leaves were sampled. Tree height was measured 23 months after planting in the three blocks (excluding three buffer rows in each subplot), and in each treatment, tree leaf area was measured destructively on eight trees per treatment at age two years to establish allometric relationships. Predawn leaf water potential, midday stomatal conductance (gs) and net CO_2_ assimilation (Asat) were measured in two-month-old fully expanded leaves at the top of the canopy on a sunny day in the same week as leaf sampling (see [23] for a description of the methodology).

### Leaf RNA sequencing

Total RNA was isolated using the modified protocol of Zeng [57]. RNA was quantified using a Nanodrop ND-1000 spectrophotometer (Thermo Fisher Scientific, Wilmington, DE, USA). Samples with a R260/280 ratio < 1.8 were discarded. A 1% agarose gel buffered by Tris–acetate–EDTA was used to determine the integrity of the RNA. RNA quality was checked using an Agilent Bioanalyzer RNA 6000 Nano kit. Samples with RNA integrity number (RIN) > 8 were selected for RNA sequencing. cDNAs libraries were prepared using TruSeq RNA Sample Prep (Illumina). Next, paired-end sequences were generated (cDNAs TruSeq PE Cluster Kit v3-cBot-HS, Illumina) and sequenced (TruSeq SBS v3-HS, Illumina, San Diego, USA) at the Functional Genomics Center (ESALQ/USP, Brazil). The four biological repetitions of the six treatments were randomly dispersed in three lanes, each lane containing eight multiplex libraries (180 million single reads per lane). Sequencing was performed on 2x101 pb on Illumina HiSeq2000 at the Esalq Genomic platform (Piracicaba, SP, Brazil). RNASeq reads were demultiplexed using Casava Software. Quality control of the reads was performed with FastQC, before and after removing the adapters with Cutadapt. The reads were trimmed if their length was < 35 bp and their PHRED Casava value was < 30. Read assembly and alignment of the *E*. *grandis* genome v1.0 (Phytozome) were performed using TopHat package v2.1.0 [58] according to the parameters listed in [59].

### Selection of significant genes

Genes from the TopHat count table with values < 1 were removed manually. Two approaches were used to extract relevant genes: differential expression and gene network analysis. Differential expression analysis was performed in two steps as described for multifactorial design (http://bioconductor.org/pack-ages/release/bioc/html/DESeq.html [60], using the DESeq2 R package [61]. Briefly, the likelihood ratio test (LRT) was applied to simultaneously test all the treatments and levels according to the multifactorial model: Fertilization + Rainfall + Fertilization x Rainfall. Significant genes were selected at FDR-corrected p-values < 0.01 threshold. This gene set, called Multifactor, represented all the genes whose level of expression changed due to the fertilization or rainfall regime, or interactions between the two. To evaluate the quality of the selected data and to explore the underlying structure of the Multifactor gene set, partial least square discriminant analysis (PLS-DA) was conducted using the mixOmics R package [62]. Next, specific differentially expressed genes (DEGs) were extracted from the Multifactor gene set to obtain a more precise understanding of the influence of the applied factors. To this end, pairwise comparison analysis was performed by applying the Wald test on the counts, between two conditions, with FDR corrected p-values < 0.01. To select genes influenced by rainfall reduction, Rainfall DEGs were extracted for each fertilization regime, according to the following comparisons: 1- K and RR vs K and FR (K Rainfall), 2- Na and RR vs Na and FR (Na Rainfall), and 3- C and RR vs C and FR (C Rainfall).

Weighted gene co-expression network Analysis (WGCNA) was performed on the Multifactor gene set using WGCNA R package [63], as described in (https://labs.genetics.ucla.edu/horvath/CoexpressionNetwork/Rpackages/WGCNA/Tutorials/). Briefly, counts of the Multifactor gene set were normalized by their relative standard deviation (RSD). They were then used to construct groups of highly correlated genes, clustered in modules based on their dissimilarity, using the following settings: power = 8, minModuleSize = 90, MEDissThres = 0.25. The relationship between the level of gene expression of each network and treatments was computed by measuring Pearson’s correlation and associated p-values. Correlations were considered significant if R > 0.70 and pval < 0.05.

### Functional annotation and classification

Blast2GO 3.0 was used to annotate Multifactor genes with the best BlastX hit in the nr database, with a E-value cutoff < 1.10^−6^ [64]. The putative orthologs of *Arabidopsis thaliana* (rate of 95%) were identified according to a high similarity *e-value* with the query. Rainfall DEGs and genes from the networks were then functionally analyzed. Gene ontology enrichment of biological processes was performed using BiNGO plugin (Cytoscape software) based on *Arabidopsis thaliana* annotation [65, 66] using default parameters. Briefly, over-representation of biological processes was assessed compared to the whole *Arabidopsis thaliana* annotation used as reference. A hypergeometric test was used as statistical test, then multiple testing correction was applied using the Benjamini & Hochberg False Discovery Rate (FDR) correction at a significance level of 0.05. To summarize and visualize enriched biological processes, map enrichment was performed. GO enrichment, generated by BiNGO [67], was used to implement the analysis using Enrichment Map plugin (Cytoscape software) [68]. Clusters of similar functional groups were then annotated using AutoAnnotate plugin (Cytoscape software) [69]. For each plugin, default parameters were applied. Enrichment analysis in the Kegg pathway were performed using David Bioinformatics Resources v6.8 [70].

### Identification of cation transporters

Potassium and sodium transporters and channels, and aquaporins were manually listed from Rainfall DEGs and Fertilization DEGs (Table 1), as well as corresponding log2Fold, representing differential expression according to the rainfall reduction, and regardless of the rainfall regime, for each fertilization regime.

**Table 1:** List of K and Na transporters, and aquaporins.

Family			Gene ID	Gene name	Log2Fold rainfall reduction			Log2Fold fertilization			At orthologs	Reference
					K	Na	C	KvsC	KvsNa	NavsC		
K channels		Shaker type	Eucgr.C01105	Voltage-gated potassium channel (AKT1)	ns	ns	ns	-0.87***	-0.87***	ns	AT2G26650	[71]
			Eucgr.L01971	Potassium outward rectifier channel (SKOR)	0.75**	ns	0.54*	0.50**	0.39*	ns	AT3G02850	[72]
		Two-pore K channels	Eucgr.F03693	Outward rectifying potassium channel protein (TPK1)	-0.35*	-0.42*	-0.47***	ns	ns	ns	AT5G55630	[73]
			Eucgr.K01218	Outward rectifying potassium channel protein (TPK3)	ns	ns	-0.35*	ns	ns	ns	AT4G18160	[74]
K transporters		KUP/HAK/KT transporters	Eucgr.B03948	Putative potassium transporter (KUP12)	0.33**	0.26*	ns	ns	ns	ns	AT1G60160	[75]
			Eucgr.B03949	Putative potassium transporter (KUP6)	ns	ns	ns	ns	0.37**	ns	AT1G70300	[17]
			Eucgr.E04300	Putative potassium transporter (KUP11)	ns	-0.39*	-0.55***	ns	ns	ns	AT2G35060	[76]
			Eucgr.E04301	Putative potassium transporter (KUP10)	ns	ns	-0.37*	ns	0.29*	ns	AT1G31120	[75]
		Putative potassium/proton antiporter	Eucgr.A02869	Potassium efflux antiporter (KEA5)	-0.39**	ns	-0.39***	ns	ns	ns	AT5G51710	[76]
			Eucgr.G01108	Putative potassium efflux antiporter (KEA3)	ns	ns	-0.39*	ns	ns	ns	AT4G04850	[77]
	Non selective cation channel (NSCCs)	Cyclic Nucleotide Gate Channels	Eucgr.A01488	Putative cyclic nucleotide-gated ion channel (CNGC1)	ns	ns	ns	-0.33***	ns	ns	AT5G53130	[78, 79]
			Eucgr.C02008	Putative cyclic nucleotide-gated ion channel (CNGC14)	ns	ns	ns	0.39*	0.31*	ns	AT2G24610	[79, 80]
			Eucgr.F03358	Putative cyclic nucleotide-gated ion channel (CNGC4)	ns	ns	ns	-0.46***	-0.34*	ns	AT5G54250	[79, 81]
			Eucgr.H00600	Putative cyclic nucleotide-gated ion channel (CNGC20)	ns	ns	ns	-0.42**	ns	ns	AT3G17700	[79, 82]
			Eucgr.I01988	Putative cyclic nucleotide-gated ion channel (CNGC2)	ns	ns	ns	-0.29*	ns	-0.46**	AT5G15410	[79, 81]
			Eucgr.K01241	Putative cyclic nucleotide-gated ion channel (CNGC15)	-0.71*	ns	-1.07***	ns	ns	ns	AT2G28260	[79]
		Glutamate-gated receptor	Eucgr.C01861	Putative glutamate receptor (GLR2)	0.93*	ns	0.99**	ns	ns	ns	AT2G29120	[83]
			Eucgr.C02006	Putative glutamate receptor (GLR2)	ns	ns	ns	-0.48**	-0.45**	ns	AT2G29120	[83]
			Eucgr.I01532	Putative glutamate receptor (GLR3)	ns	ns	ns	-0.59**	ns	ns	AT4G35290	[84]
			Eucgr.I02216	Putative glutamate receptor (GLR3)	0.45*	ns	ns	ns	ns	ns	AT3G51480	[85]
			Eucgr.K00799	Putative glutamate receptor (GLR3)	0.46*	ns	-0.38*	ns	ns	ns	AT1G05200	[83]
			Eucgr.L03706	Putative glutamate receptor (GLR2)	0.90*	ns	1.03**	ns	ns	ns	AT4G31710	[86]
	Na transporter	HKT transporter	Eucgr.C02181	Sodium transporter (HKT1)	ns	ns	ns	-0.36**	-0.43***	ns	AT4G10310	[87]
Sodium/proton exchanger			Eucgr.B01758	Sodium/H+ exchanger (NHX2)	ns	ns	-0.48**	ns	ns	ns	AT3G05030	[88]
			Eucgr.D00309	Putative cation/H+ exchanger (CHX4)	-0.90*	ns	-0.80*	ns	ns	ns	AT3G44900	[89]
			Eucgr.E04240	Sodium/H+ exchanger (NHX6)	ns	ns	-0.38**	ns	ns	ns	AT1G79610	[89]
			Eucgr.H04454	Sodium/H+ exchanger (NHX2)	ns	ns	ns	0.48***	0.48***	ns	AT3G05030	[90]
Cation/proton exchanger			Eucgr.A00502	Cation/H+ exchanger (CAX3)	ns	ns	ns	-0.50*	ns	-0.54*	AT3G51860	[91]
			Eucgr.A02141	Cation/H+ exchanger (CAX1)	ns	ns	ns	-0.77***	-0.79***	ns	AT5G17860	[91]
Water channel		Aquaporins	Eucgr.A01153	Aquaporin PIP1-3/PIP1-4	ns	ns	0.48***	-0.61***	-0.63***	ns	AT2G37170	[92]
			Eucgr.A02176	Aquaporin SIP2-1	ns	ns	-0.52***	ns	ns	ns	AT3G56950	[93]
			Eucgr.D00421	Aquaporin NIP1-1	ns	ns	ns	ns	0.62***	-0.68***	AT4G18910	[92]
			Eucgr.F03054	Probable aquaporin TIP-type	ns	ns	ns	-0.31*	-0.35**	ns	AT3G16240	[94]
			Eucgr.G03037	Aquaporin PIP1-3/PIP1-4	ns	ns	-0.41**	ns	ns	ns	AT4G00430	[92]
			Eucgr.I01369	Aquaporin PIP2	-0.47*	ns	ns	ns	ns	ns	AT4G00430	[92]
			Eucgr.J00930	Aquaporin PIP1-3/PIP1-4	ns	-0.50**	0.44***	ns	0.70***	-0.47**	AT5G60660	[92]
			Eucgr.J01087	Aquaporin PIP2	-0.55**	ns	ns	ns	ns	ns	AT3G54820	[92]
			Eucgr.J01345	Aquaporin NIP2	-0.78*	ns	ns	0.55*	0.62**	ns	AT5G37820	[92]

## Results

### Ecophysiological parameters

**Fig 1:**
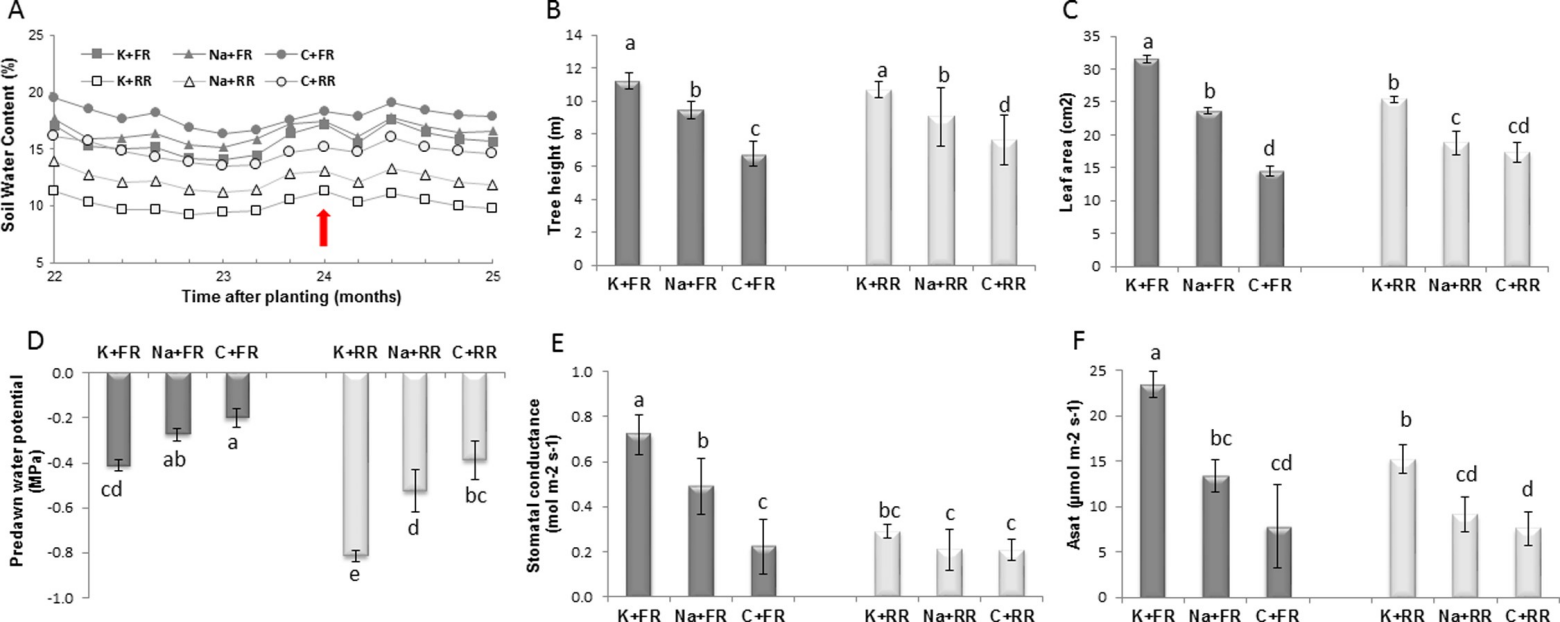
Effect of a 37% reduction in rainfall and K and Na fertilization regimes on soil water content and eucalyptus physiological parameters. Mean soil water content (in the 0.15 to 6 m soil layer), 22 to 25 months after planting. The leaf sampling date, at two years of age, is represented by a red arrow (A). Measurements on two-year-old trees of mean tree height (B), leaf area (C), predawn leaf water potential (D), midday stomatal conductance gs (E) and CO_2_ assimilation rate (F) measured on two-month-old leaves. Dark gray = full rainfall; Light gray = reduced rainfall.

Soil water contents (SWCs), measured from 22 to 25 months after the trees were planted, were mainly influenced by the water supply regime (Fig 1A). Mean SWC in the 0–6 m soil layer was much higher in FR (17.2%) than in RR (12.8%) and was influenced by the fertilization regime. At 24 months after planting, SWCs were lowest under the two rainfall regimes in K-fertilized plots (16.6% and 10.7% on average in FR and RR, respectively), while C plots had the highest SWCs (18.3% and 15.2% in FR and RR, respectively). In Na-fertilized plots, SWCs were similar to those in K-fertilized plots with FR (17%), and intermediary between C and K with RR (12.7%).

K and Na fertilization positively influenced tree height (52% and 28% higher than in C treatment, respectively) and leaf area (28 and 21% higher than in C treatment, respectively), (Fig 1B and 1C). Tree leaf area was only affected by RR in K- and Na-fertilized trees, with a decrease of about 20% relative to FR. RR led to a significant decrease in pre-dawn leaf water potential under both fertilization regimes compared to FR (Fig 1D). Pre-dawn leaf water potential was, respectively, 2 and 1.3 times lower in K- and Na-fertilized trees compared to control trees. RR significantly affected the midday stomatal conductance of K- and Na-fertilized leaves (respectively 67% and 57% lower than in FR) (Fig 1E), and CO_2_ assimilation rates (about 37% lower than in FR for K-and Na-fertilized trees) (Fig 1F).

### Rainfall reduction regulated leaf transcriptome differentially with K or Na fertilization

**Fig 2:**
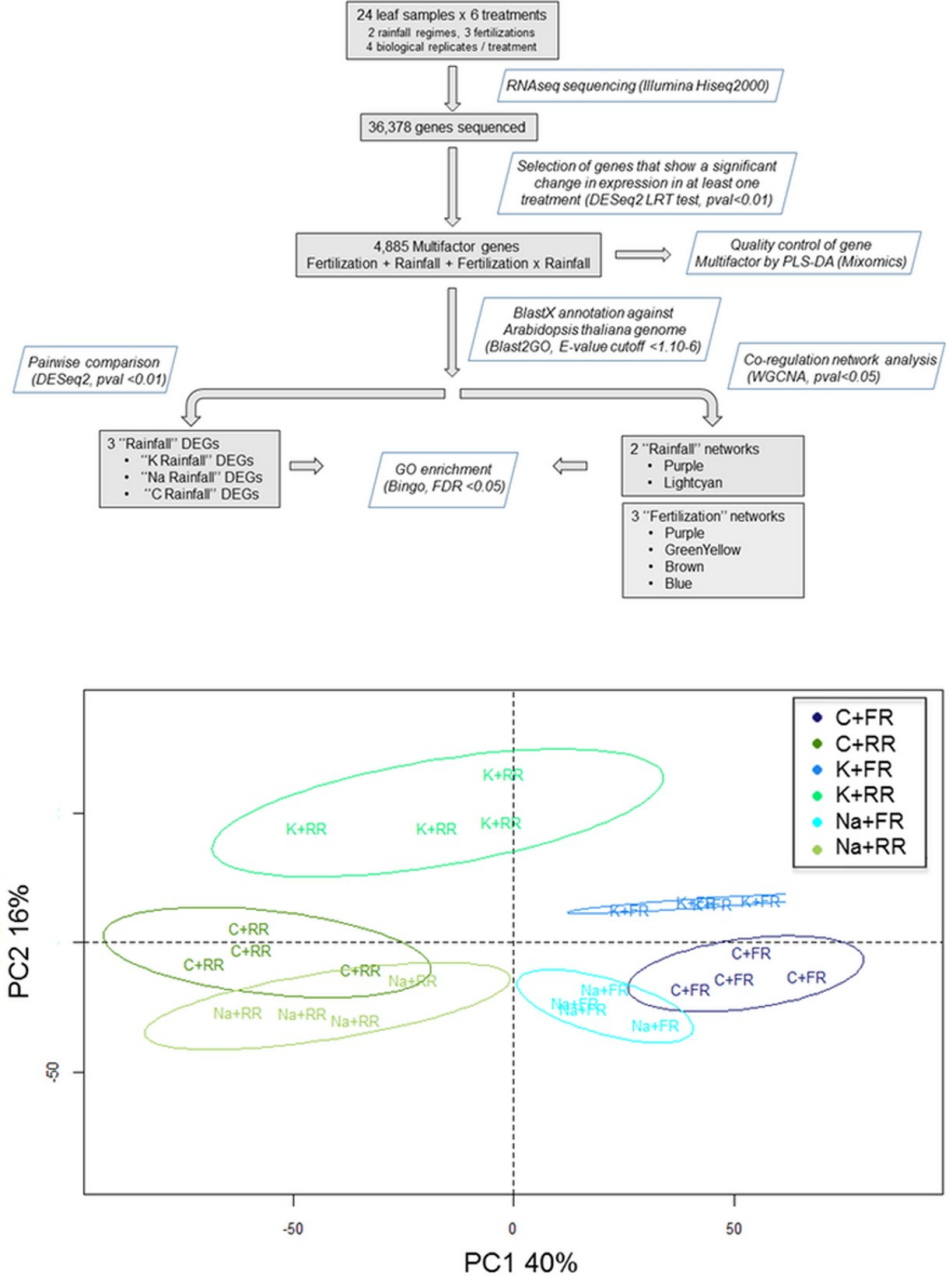
Selection of significant genes. (A) Flowchart showing the data selection process. (B) PLS-DA of 4,885 Multifactor DEGs on the first two components.

Following the read sequencing, and sequence alignment and assembly, 36,378 genes were extracted. From this dataset, a specific strategy using LRT test was applied to select 4,885 genes showing a significant change in expression in at least one treatment (FDR < 0.01, no log2Fold cutoff), and latter referred to Multifactor genes (Fig 2A and S1 Table). PLS-DA was performed on Multifactor genes to evaluate the quality and the structure of the data selection. The first four principal components (PC) explained 73% of total variability, with PC1 distinguishing conditions FR and RR (40%), and PC2 the K treatments, on one hand, and C/Na, on the other hand (16%) (Fig 2B). Overall, we showed that (i) the four biological replicates were correctly classified according to the treatments they had received, (ii) the first factor driving transcriptomic leaf response was rainfall availability, (iii) response to the rainfall regime was strongly dependent on the fertilization regime. Consequently, the Multifactor gene set explained the variability between the treatments and can be used to analyze the biological processes involved in the response to rainfall reduction in interaction with the fertilization regime.

**Fig 3:**
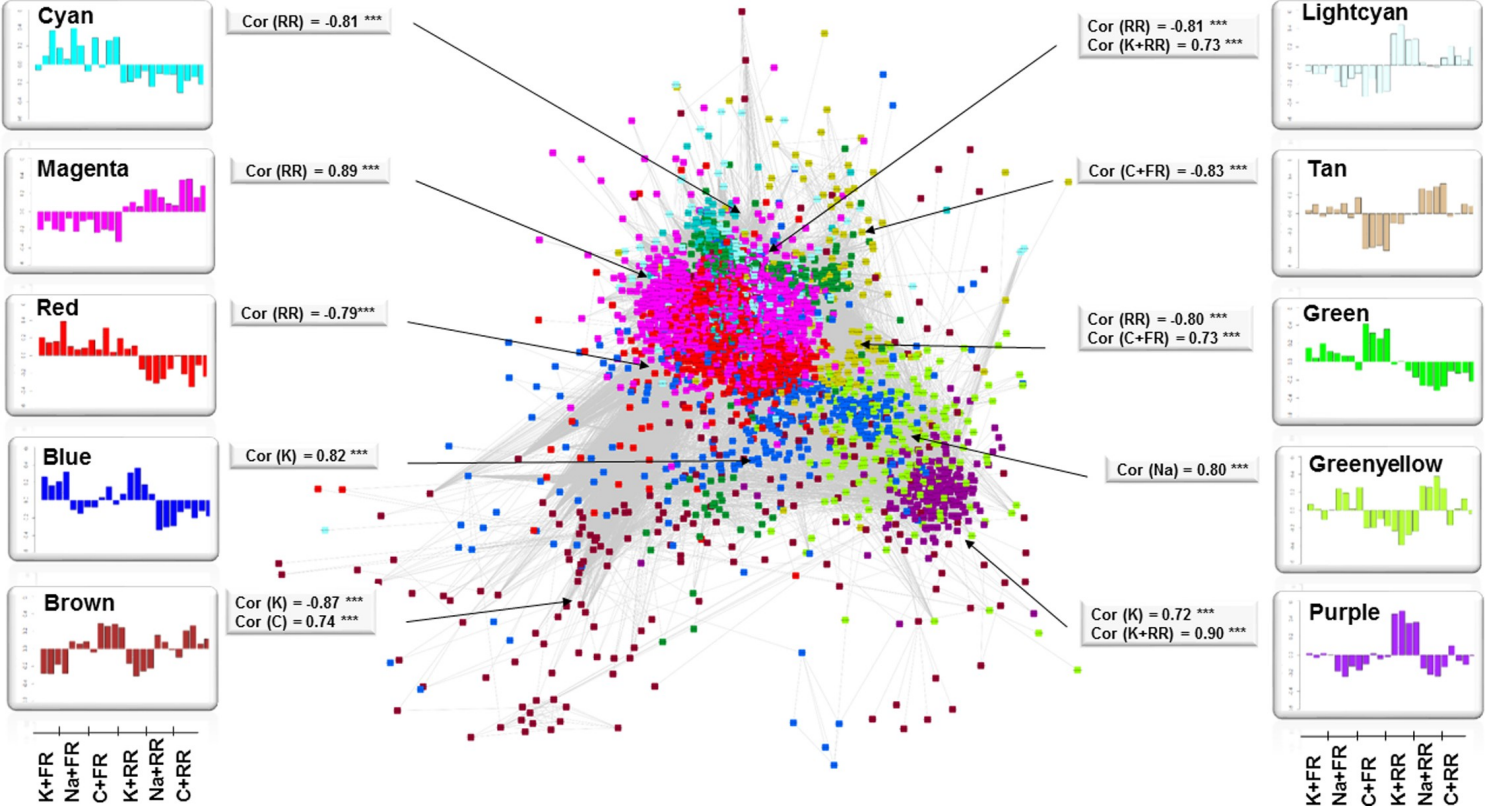
Representation of the 10 network modules and corresponding bar plots representing the gene expression level for each treatment, with four replicates per treatment. The number of genes (n), the most significant R correlation values (Cor > 0.70 and pval > 0.05) and the corresponding level of significance (ns > 0.05; *< 0.01; **< 0.001; ***< 0.0001) per treatment are given for each module. Gene expression was significantly affected by reduced rainfall (RR = Cyan, Magenta, Red, Lightcyan, Green), potassium and reduced rainfall (K and RR = Lightcyan, Purple), control and full rainfall (C and FR = Tan, Green), potassium (K = Blue, Brown, Purple), sodium (Na = GreenYellow), control (C = Brown).

Two strategies were applied to measure this response. On one hand, pairwise comparison analysis was performed on Multifactor DEGs to extract significant genes that responded to rainfall reduction under each fertilization regime, hereafter referred to as Rainfall DEGs. On the other hand, Multifactor DEGs were used to build a gene co-regulation network based on weighted pairwise correlation between gene expression (p-value < 0.05) using WGCNA protocol. Ten modules were detected containing highly correlated genes, and therefore potentially involved in the same biological process. For each module, a corresponding heatmap presents the mean gene expression level for each sample (Fig 3). Correlation analysis was performed to test the biological significance of the gene expression level under each treatment. Correlations were considered significant when absolute values were > 0.70 and the corresponding p-value < 0.05 (S2 Table). To analyze the response to rainfall reduction in interaction with the fertilization regime, and to fertilization regardless of rainfall reduction, modules selected were: Lightcyan and Purple correlated with K and RR; Blue, Purple, Brown correlated with K; GreenYellow correlated with Na; Brown correlated with C.

### Effect of rainfall reduction on leaf transcriptome

**Fig 4:**
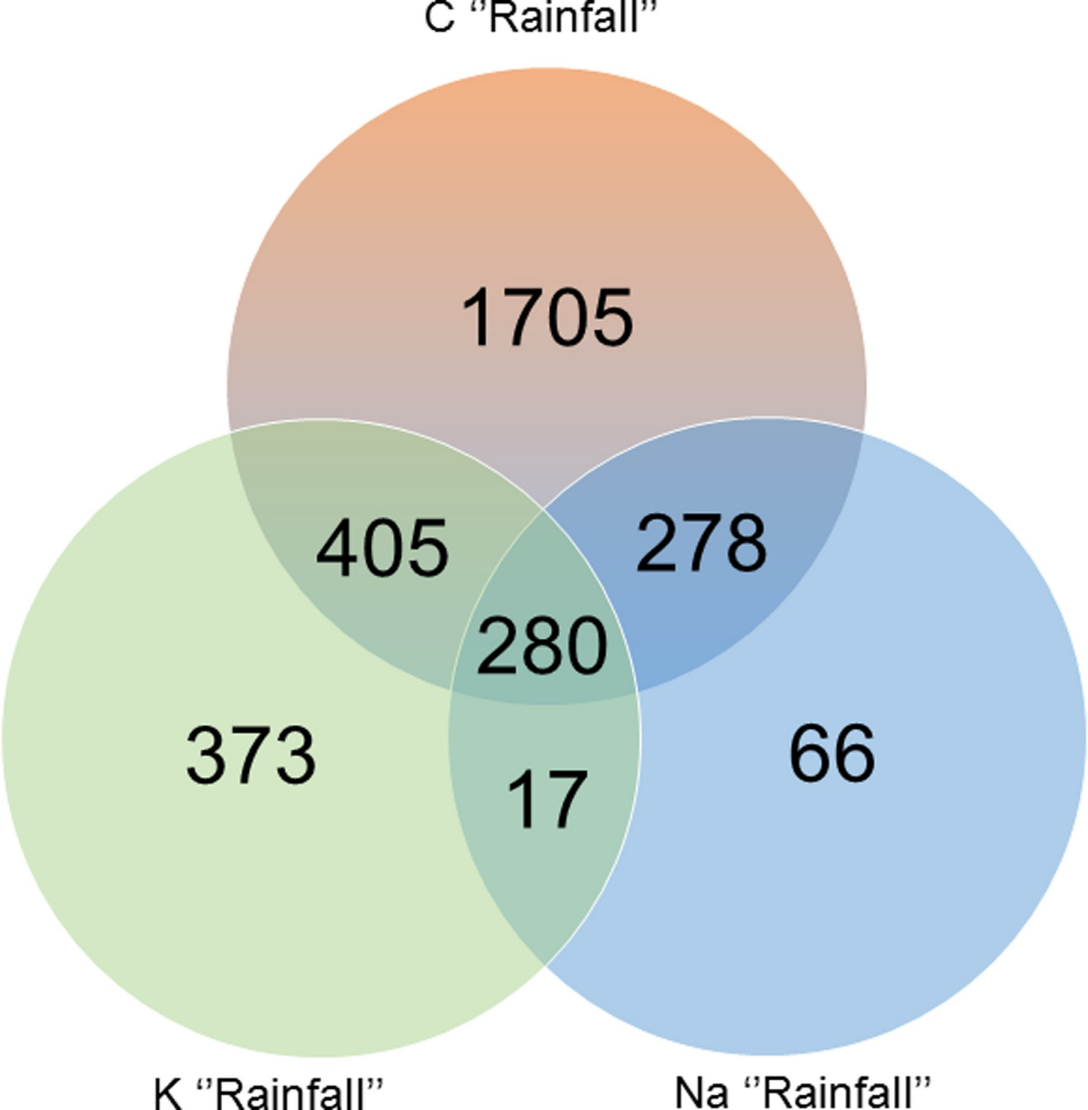
Venn diagram of Rainfall DEGs.

**Fig 5:**
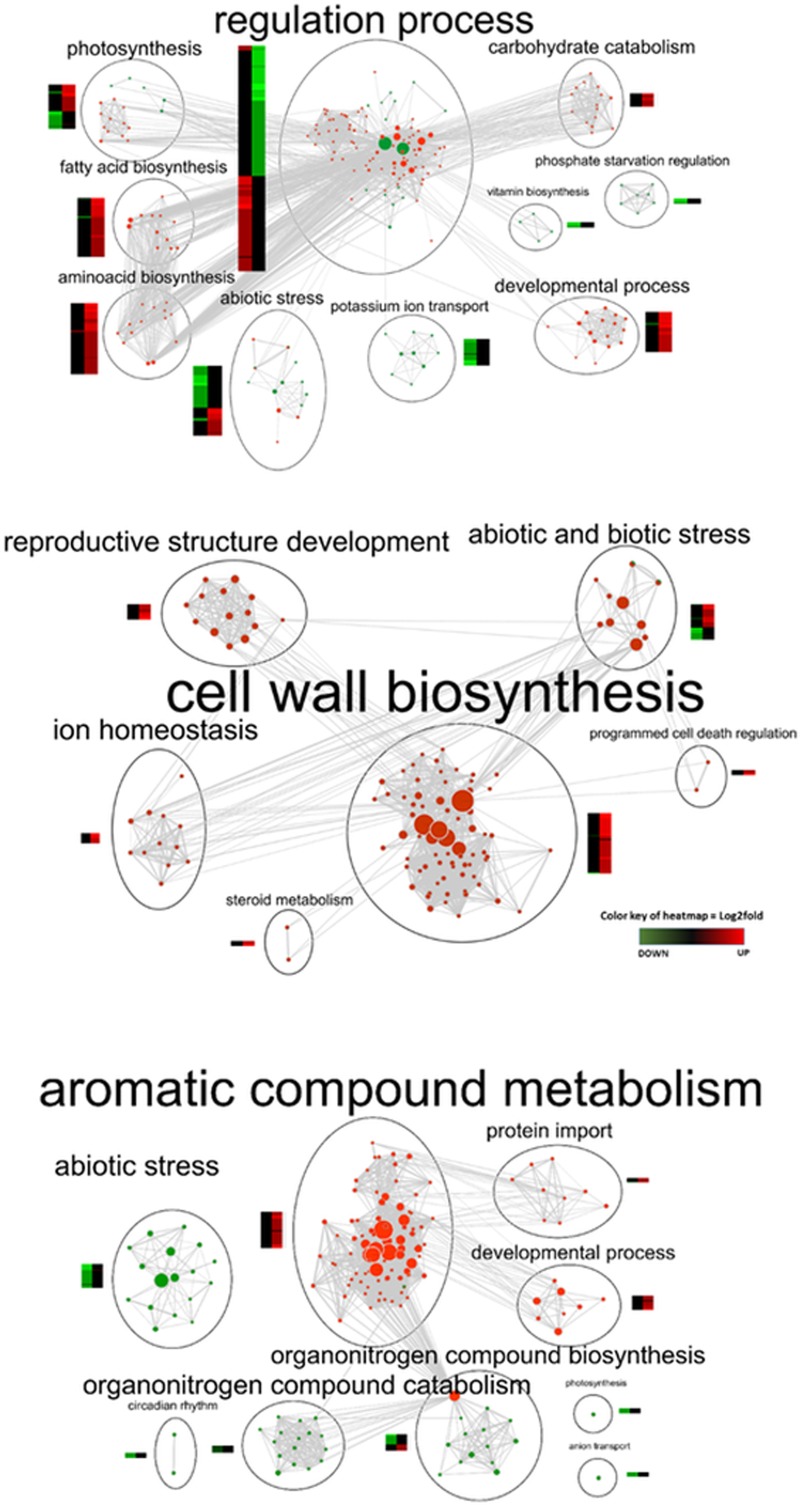
**Enrichment map of genes expressed under the Reduced Rainfall treatment with no fertilization (A), with K fertilization (B), with Na fertilization (C).** The map includes nodes representing GO enriched gene sets connected by their edges, representing similarity between two gene sets. Nodes belonging to very similar biological processes were clustered and labeled with a summarized name. For each cluster, the heatmap of up- and down-expressed genes in response to RR is shown. Enrichment significance (p-value) is conveyed by the node color in the corresponding up and down-expressed genes; enrichment significance (p-value) is conveyed by the size of the node, which is proportional to the number of up-and down-expressed genes; the edge thickness is proportional to the degree of similarity between two nodes. HeatMap: the color represents positive and negative log2 fold changes in gene expression.

The Venn diagram comparing the three sets of genes differentially expressed in response to RR Rainfall DEGs, in the three fertilization treatments (Fig 4), revealed that the K-deficient condition C Rainfall had the highest number of unique genes (55%), compared to the K and Na fertilized treatment, while Na Rainfall had the lowest one (2%). Only 9% of genes regulated under RR are common to the three fertilization regimes, and less than 1% were shared by K and Na. To gain further insights into the regulation of leaf transcriptome, functional analysis was performed on the three Rainfall DEGs to identify the biological processes regulated in response to RR in interaction with fertilization. Detailed results of GO and KEGG pathway enrichments are presented in supporting information: S3 Table of K Rainfall, Na Rainfall and C Rainfall DEGs, and S2 Table for Lightcyan, and Purple networks. The results for each gene set are summarized as an enrichment map (Fig 5). GO enrichment in response to rainfall reduction is detailed below for each fertilization regime.

#### Effect of RR on leaf transcriptome from K-deficient trees

A GO enrichment analysis was performed on the C Rainfall subset of genes significantly regulated by rainfall reduction, in non-fertilized leaves (S3 Table). Results are summarized in Fig 5A. Regulation processes involved post-transcriptional regulation (139 genes) comprising transcription activity, mostly activation of DNA-templated RNA polymerase and formation of heterochromatin (17 genes), a well as the production of siRNA (5 genes). Translation (86 genes), and post-translational modifications and protein synthesis were also regulated with ncRNA metabolism, mostly tRNA and rRNA (23 genes), protein complex assembly (16 genes) and protein folding (30 genes). Protein domain analysis revealed an increase in chaperons, chaperonins and heat-shock proteins involved in protein folding. Biological processes related to modifications in primary metabolism were also identified, including carbohydrate catabolism (17 genes) and starch catabolism (7 genes), nitrogen compound (oxoacid metabolism: 52 genes; amino acid metabolism and biosynthesis: 34 genes and 17 genes; amino acid and derivatives: 41 genes), and fatty acid (oxoacid metabolism: 52 genes; fatty acid biosynthesis: 12 genes). Several processes related to photosynthesis were regulated including the photosynthetic electron transport chain (6 genes down-regulated), photosynthesis light reaction (9 genes down-regulated), phosphorus metabolism (61 genes down-regulated), plastid organization (21 genes up-regulated), protein complex assembly (11 genes up-regulated), photosystem I assembly (3 genes up-regulated). Up-regulation of a subset of genes was related to developmental processes including modification of anatomical structure (111 genes) and reproductive structure (67 genes), plus embryonic development (67 genes) and seed development (51 genes). An induction of genes involved in abiotic stress response was also detected, included salt stress (28 and 30 genes up- and down-regulated) and water deprivation (17 genes down-regulated). Down-regulation of peptide and ion transport (94 genes) was also observed, comprising seven potassium ion transporters, as well as phosphate starvation (3 genes).

#### Effect of RR on leaf transcriptome from K-fertilized trees

**Fig 6:**
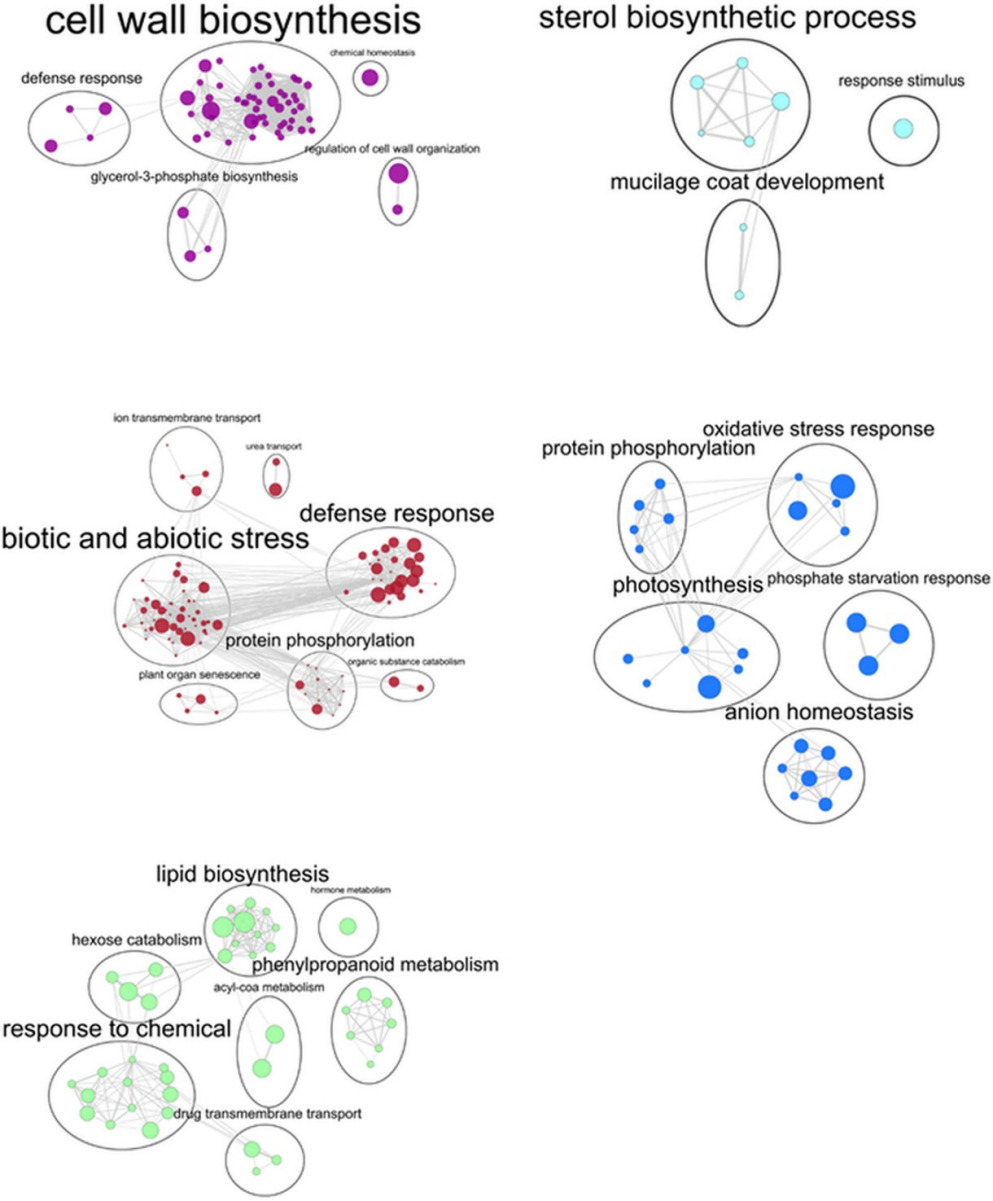
Enrichment map of network modules positively (+) or negatively (-) correlated with treatments (pval < 0.05; R > 0.7). (A) Purple = K and RR and K (+); (B) Lightcyan = K and RR (+); (C) Brown = C (+), K (-); (D) Blue = K (+); (E) GreenYellow = Na (+). Node = GO enrichment of gene set; Node size is proportional to significance (p-value); Edge is the overlap between two connected gene sets; Edge thickness is proportional to the amount of overlap.

GO enrichment of K Rainfall highlighted secondary cell wall biogenesis as the main biological process that increased when RR was compared to FR (S3 Table and Fig 5B). This response was also detected in the Purple network, representing gene expression in response to K and RR (Fig 6A). Up-regulation of gene expression involved in primary metabolism led to synthesis of high molecular weight compounds involved in cell wall formation, including polysaccharides (12 genes), cellulose (7 genes) and lignins (41 genes). The Kegg pathway displayed increased transcriptional activity, although to a much lesser extent than in the control treatment, involving ribosome biogenesis (14 genes) and pyrimidine metabolism (8 genes). Protein folding (14 genes up-regulated) was confirmed by an increase in WD-40, chaperonins and heat shock proteins. Stress responses included regulation of temperature (16 genes up-regulated), and water deprivation (26 genes up- and down-regulated). Increased response to biotic stress was detected (24 genes) including regulation of programmed cell death (4 genes) and defense against fungus (7 genes). Like in the control treatment, the expression of some genes involved in the development of the reproductive structure was up-regulated (27 genes), including seed and fruit development (19 genes) and steroid metabolism (6 genes). Like the response observed in K-deficient trees, increased ion homeostasis (7 genes) was observed, comprising four putative orthologs of glutamate receptor transcripts acting as non-selective cation channels and potassium transport.

#### Effect of RR on leaf transcriptome from Na-fertilized trees

In the Na Rainfall gene set, few biological processes were affected by rainfall reduction, compared to those detected in C Rainfall and K Rainfall (S3 Table and Fig 5C). Regulation of gene expression (42 genes) included over-expression of genes involved in ncRNA metabolism (20 genes), RNA processing (24 genes), RNA modification (14 genes). Protein folding was over-regulated (9 genes). The analysis highlighted the overall regulation of nitrogen (66 genes) and aromatic compound metabolism (58 genes). Over-representation of genes involved in aromatic compound biosynthesis (18 genes) involved amino acid (8 genes) and pyrimidine biosynthesis (6 genes), with de-novo processes (2 genes). Transcripts corresponding to organonitrogen biosynthesis and catabolism were down-regulated, including nucleobase metabolism (6 genes), oxoacid metabolism (25 genes), sulfur amino acid biosynthesis (5 genes), aromatic compound catabolism (9 genes), flavonoid biosynthesis (4 genes). A group of genes related to developmental processes was activated along with leaf development (10 genes), embryo development (16) and megagametogenesis (4 genes). Only a small fraction of mainly down- regulated genes were related to photosynthesis (10 genes) and anion transporter (10 genes). Transcripts classified as abiotic stress responses were down-regulated (49 genes) with genes involved in water deprivation (12 genes), osmotic stress (17 genes), response to light (20 genes), and to chemicals (51 genes).

### Effect of different fertilization regimes on leaf transcriptome

Co-regulation network analysis detected four modules of highly correlated genes whose gene expression level was significantly influenced by K and Na fertilization (Supplementary S2 Table). Functional analysis was performed as explained above, with analysis of GO and Kegg pathway enrichment (S2 Table). Results are summarized in an enrichment map (Fig 6) and described below.

The Brown module was positively correlated with the control treatment. Enriched biological processes in the Brown network pointed to a very significant response to stress (75 genes) (Fig 6C), with genes responding to salt stress and osmotic stress (18 and 19 genes), negative regulation of response to water deprivation (2 genes), and response to starvation (8 genes). Genes related to response to biotic stress also increased in this dataset, including defense response (14 genes), hormonal responses (36 genes), cell surface receptor signaling pathways (13 genes) and positive regulation of defense responses (8 genes). Protein phosphorylation was one of the main processes involved in the regulation (40 genes). Ion transport was also regulated (8 genes), including two K transporters. Plant organ senescence comprised genes involved in aging (7 genes), and regulation of leaf development and senescence (4 genes).

Two networks, Purple and Blue, were positively correlated with the response to K fertilization (Fig 6A and 6D). The Purple network comprised genes involved in cell wall biosynthesis (11 genes), carbohydrates (13 genes), glucan (8 genes), and cellulose (6 genes). Ion homeostasis was detected (4 genes), with three genes involved in potassium transport, and defense responses (6 genes). The Blue network comprised genes involved in photosynthesis (12 genes) along with protein phosphorylation (34 genes) and response to oxidative stress (16 genes). Phosphate regulation comprised anion homeostasis (6 genes) and phosphate starvation regulation (2 genes). In addition, the biological processes in the Brown network described above were negatively correlated with K fertilization.

The GreenYellow network was positively correlated with Na fertilization (Fig 6E). Corresponding genes belonged to biological processes related to lipid metabolism (22 genes), as well as secondary metabolism (12 genes) including phenylpropanoid biosynthesis (7 genes), lignin metabolism, and response to toxic substance (7 genes). A response to chemicals (40 genes) was detected, related to drug transmembrane transport (5 genes).

### Identification of cation and water transporters

In the Multifactor gene set, 29 putative orthologs of K channels and transporters were manually identified, these included sodium/proton exchangers and cation/proton exchangers, as well as 9 aquaporins involved in water transport (Table 1). The K transporter family comprised 12 genes: 3 Shaker type, 2 two-pore K channels, 4 KUP/HAK/KT transporters, 2 putative K/H+ antiporters, and 1 HKT transporter. Twelve genes belonged to the non-selective cation channel (NSCC) family, which includes 6 cyclic nucleotide gate channels (CNGCs), and 6 glutamate receptors. Six genes were involved in cation transport comprising 4 sodium/proton exchangers and 2 cation/proton exchangers. Among all the K and Na transporters, 18 were referenced as putative genes. The aquaporins belonged to several families: 7 PIPs, 2 NIPs, 1 TIP-type, and 1 SIP.

Table 1 legend. List of K and Na transporters, and aquaporins differentially expressed as a function of the rainfall reduction and fertilization treatments with their respective log2 fold change and p-values (ns > 0.01; *< 0.01; **< 0.001; ***< 0.0001). Corresponding *Arabidopsis thaliana* ortholog and article references are given for each gene.

Differential expression of these genes was measured according to rainfall exclusion, on one hand, and fertilization, on the other. Sixteen genes acting as selective and non-selective K and Na transporters were regulated as a result of rainfall reduction, with six out of 10 over-expressed with K fertilization, and eleven out of 14 down-expressed in the control treatment. Only three genes were differentially expressed with Na fertilization, KUP12 positively, and TPK1 and KUP11 negatively. Fifteen were regulated as a function of fertilization. Only three genes were over-expressed with K, compared with Na and C (SKOR, CNGC14, NHX2), and two compared to Na alone (KUP6, KUP10). Only two genes were differentially and negatively expressed with Na compared to C (CNGC2, CAX3).

Nine aquaporins were regulated as a function of rainfall reduction, including five PIPs, one NIP and one SIP. All were down-expressed with K (3 genes) and Na (1 gene) fertilization, while two up- and two down-expressed were detected with the control treatment. The five aquaporins regulated by fertilization differed from the ones identified with rainfall reduction, and comprised two PIPs, two NIPs and one TIP. No distinct expression pattern was identified.

## Discussion

The aim of this study was to measure the effect of K and Na supply on the global leaf transcriptomic response in water-deficient *Eucalyptus grandis*. Analysis of data resulting from a multifactorial experimental design required the use of a specific strategy to disentangle the combined effect of water and fertilization regimes. First, multivariate statistical approaches enabled selection of the genes showing a significant change in expression under at least one treatment. The resulting data structure revealed that, at the transcriptomic level, leaf metabolism mainly responded to RR, in contrast to previous observations made at tree level [23]. Moreover, the leaf response to RR was highly dependent on the fertilization regime. To extract the genes responding to water deficit and fertilization on one hand, and to fertilization regardless of the rainfall regime, on the other hand, two independant statistical methods were applied, standard pairwise comparison and weighted correlation network analysis (WGCNA). WGCNA, which was originally developed for microarray datasets and transcriptomic profiling experiments, allowed us to identify genes with more informative biological meaning [95, 96]. WGCNA enabled us to: 1) detect associations between treatments and groups of correlated genes, 2) validate highly significant molecular processes identified by pairwise comparison analysis, and 3) identify weaker but nevertheless significant biological signals. WGCNA was particularly useful to identify processes related to Na fertilization, which was previously shown to drive an intermediate response between K deficiency and K fertilization [44]. This overall strategy provided very informative gene lists that were then functionnally analyzed. Contrasted molecular processes in response to rainfall reduction and/or fertilization were revealed and are described below, in relation to tree and leaf physiological responses in the field. To exploit findings from past research, we built new hypothesis to elucidate the eucalyptus leaf response to water deficiency combined with K and Na supply.

### Stress response

Eucalyptus leaf physiology was affected by rainfall reduction (RR), with a similar decrease in the level of dehydration (about -90%), in response to RR under all fertilization regimes, compared to the initial level under full rainfall (FR). However, midday stomatal conductance and CO_2_ assimilation decreased more in K-deficient leaves than in K- and Na-fertilized trees. K-deficient leaves displayed the strongest response to stress caused by RR, with increased post-transcriptional and post-translational regulation, and protein folding including HSPs activity. In plants, post-transcriptional and post-translational regulations are known to be involved in abiotic stress [97, 98]. HSPs are involved in protein folding as well as in responses to drought and heat stress [99]. In poplar leaves, strong HSP induction revealed a drought tolerant strategy [100]. The regulation of responses to high stress detected in K-deficient eucalyptus leaves was accompanied by complex up- and down-regulation of salt stress and water deprivation responses. Moreover, response to salt stress was positively correlated with K deficiency regardless of RR. In plants, both K- and water deficiency severely affect metabolism, including growth and development, and lead to the regulation of many common genes, resulting in complex cross-talk and interactions [101]. In K-deficient leaves, stress response involved 20 transcription factors, including 10 MYB and MYB-related families known to be involved in the regulation of plant stress responses [102]. Other transcription factors were detected, including ABF3 (abscisic acid responsive element-binding factor 3) and ABA2 (ABA deficient 2), known to be involved in ABA regulation or in ABA-responses, and regulated under drought, salt stress, and ABA [103–105]. ABA plays an important role in signaling, abiotic stresses such as salt and drought stress [106], and has been shown to accumulate in the roots and leaves of K-deficient plants [107]. Transcription factors that belong to ABA-dependent and ABA-independent pathways are known to be involved in rapid or adaptive response [108, 109].

In K-supplied leaves and RR, a weaker stress response was detected than in K-deficient leaves, with less protein folding. Furthermore, a weak but positive response to water deprivation was revealed. Fewer transcription factors were regulated, including some ABA-responsive genes such as OCP3 and HB7. OCP3 (over-expressor of cationic peroxidase 3) plays a pivotal role in the signal pathway that controls drought tolerance through the modulation of ABA-mediated stomatal closure in Arabidopsis [110]. The probable transcription factor HB7 (homeobox 7) acts in an ABA-dependent manner to regulate growth in response to drought in *A*. *thaliana* and peanut [111, 112]. Water deprivation and salt stress responses were down-regulated in Na-fertilized leaves and RR, and 90% of the genes were similar to those detected in the case of K deficiency. Protein folding was as up-regulated with K fertilization. Taken together, these results revealed that stress responses to RR were highly contrasted among the fertilization regimes. K-deficient leaves displayed increased regulation of stress response and response to salt stress, the latter being up-regulated regardless of the rainfall regime, and down-regulation to water stress. K-fertilized leaves responded to water deprivation, while Na-fertilized leaves responded to water deprivation and salt stress through down-regulation of their responses. No specific stress-related genes were revealed with Na supply.

### Photosynthesis

In eucalyptus leaves, photosynthesis was affected at the molecular and physiological level by both RR and the fertilization regime. K-deficient leaves had the lowest stomatal conductance and CO_2_ assimilation, whatever the rainfall regime. The low stomatal conductance and CO_2_ assimilation observed with RR was mitigated with K supply, whereas stomatal conductance only decreased significantly in Na-fertilized leaves. Similarly, photosynthetic-related genes were down-regulated in leaves with K deficiency and, to a lesser extent, in Na-fertilized leaves. Conversely, photosynthetic-related genes were over-expressed in K-fertilized leaves. In plants, water stress affects photosynthesis by reducing stomatal activity, CO_2_ absorption [113, 114], and/or by adapting their photosynthetic metabolism [115]. A decrease in photosynthetic activity and down-regulation of photosynthetic-related genes has been reported in leaves sampled from drought-stressed loblolly pine and poplar [116, 117]. In drought-stressed olive leaves, the decrease in stomatal conductance and CO_2_ assimilation observed in K-deficient soils, was reported to be lower when trees were fertilized with K and Na [37]. Potassium regulates photosynthesis at many levels, including ATP synthesis, activation of enzymes involved in photosynthesis, CO_2_ uptake, the balance of the electric charges required for photophosphorylation in chloroplasts, and acts as the counter ion to light-induced H^+^ fluxes across thylakoid membranes [10]. These results confirmed the beneficial effect of K on photosynthesis observed in the field. The intermediary response of Na-fertilized leaves has also been observed in drought-stressed olive leaves, whose normal assimilation capacity was reported to be preserved but not stomatal conductance [37]. These results suggest that this ion is not as effective as K in regulating cell turgor. Guard cells appear to be equipped with a Na uptake system, but, depending on the plant species, stomatal regulation could be limited by Na release [39, 118–121], because of the selectivity of ion transport system [39]. In our data set, we found no differential expression of ion channels regulated by K, such as GORK, the only certain candidate mediating stomatal closure [122], or non-selective cation channels (NSCCs) that have been shown to be involved in guard cell regulation [123] (see Table 1). Other factors such as ABA signaling, may be involved in regulating guard cells [124]. Further studies are required to improve our understanding of the mechanisms involved in photosynthetic activity in relation with low Na supply.

### Primary and secondary metabolism

In eucalyptus leaves, carbohydrate metabolism was found to be modulated under RR and K-deficiency, involving starch and glucan catabolism, glycolytic process, along with aminoacid and fatty acid biosynthesis. Similarly, other studies have shown that plants subjected to drought and/or K-deficiency, and with lower photosynthetic activity, reduced starch and glucan biosynthesis, and increased sugar and amino acid biosynthesis, revealing a disturbance in carbohydrate metabolism through down-regulation of glycolysis [100, 125–127]. The glycolytic process enables modulation of carbon metabolism in response to long-term adaptive changes to environmental stresses, such as nutrient limitation and drought, with a significant proportion of the carbon used for the biosynthesis of numerous compounds, including isoprenoids, aminoacids, nucleic acids, and fatty acids [128]. Nitrogen metabolism was affected in Na-fertilized leaves and RR, with complex regulation of pyrimidine biosynthesis, aminoacid and aromatic compound metabolism. This modification of nitrogen metabolism was also observed in plants subjected to abiotic stresses which tend to accumulate nitrogen-containing compounds [129–131], including the free aminoacids proline or glycine, which act as osmolytes produced under different types of stress [132–134]. No networks were found to be correlated with Na and RR, unlike in K-deficient and K-fertilized leaves, which could have revealed specific molecular processes. This suggests that leaf response to Na fertilization involves fine regulation of metabolism, in agreement with the intermediairy response between K-deficiency and K-fertilization detected by Battie-Laclau [23]. To the best of our knowledge, the effect of low sodium fertilization on primary and secondary metabolism, and its interaction with drought conditions, has not yet been deciphered. Connections and divergences between Na nutrition and toxicity may exist, but remain to be investigated, especially at molecular level [135].

### Leaf cell wall

In eucalyptus leaves, cell wall structure was affected by the supply of both K and Na. At the physiological level, turgor in K- and Na-fertilized leaves increased due to a reduction in osmotic potential caused by water deficit [136], and only K-fertilized leaves displayed increased cell wall rigidity [23]. At the molecular level, secondary cell wall biosynthesis was triggered by K fertilization interacting with RR, and regardless of RR, with up-regulation of monolignol formation, cellulose biosynthesis, including genes (IRX3, CesA4, IRX1) more essential for cellulose synthesis in secondary cell walls [137]. Arabidopsis mutants defective in these genes have thinner secondary cell walls, which have been shown to either improve or impair resistance to biotic and abiotic stresses, depending on the plant species [138, 139]. Potassium can affect leaf morphology including specific leaf area, density and thickness [56, 140, 141]. K-deficient plants can present symptoms of wilting, due to disturbed water balance and limited lignification of their cell walls [142], while K-supplied plants may have thicker cell walls [11]. Increasing cell wall rigidity or elasticity is one of the strategies used to maintain leaf turgor under dehydration [143, 144]. With a supply of Na, leaf cell wall structure was regulated regardless of the rainfall regime. Up-regulated genes were involved in secondary cell wall biosynthesis, although to a lesser extent than with K supply, and were also involved in wax formation. In Arabidopsis, an increase in the amount of wax and in epidermal cell wall thickness was shown to be correlated with over-expression of genes associated with cuticle production [145]. Cuticles play an important role in preventing water loss in drought conditions or under salt stress [146–148]. These results suggest specific mechanisms in K and Na-fertilized leaves to prevent cell wall dehydration, activated under water deficiency with K fertilization, and independently of the rainfall regime with Na fertilization.

### Contrasted patterns of cation transporters and aquaporins were revealed in relation with rainfall reduction and fertilization

In our study, 30 cation transporters were found to be differentially regulated, including 18 putative ones. Contrasting profiles were observed depending on RR and on the fertilization regime, and on fertilization regardless of the rainfall regime. With RR, the genes belonged to all families except the cation/exchanger, while more non selective cation channels (NSCCs) were regulated as a function of the fertilization regime. In plants, membrane transporters control ion homeostasis, and play a role in plant adjustment to drought [149]. K transport has been mainly studied in the roots from which K is taken up, whereas the majority of K ions are found in the leaves and stem [124]. In leaves, specific transporters are coordinated between different compartments, including mesophyll, epidermis, and guard cells [124].

With RR, most of the genes in K-deficient leaves were down-regulated, in line with complex metabolic regulation in response to interactions between K depletion and rainfall reduction. Conversely, K transport increased with K fertilization, while weak regulation of K transport was detected with Na fertilization. While drought-stressed plants require more K, K uptake has been shown to decrease under drought due to a reduction in ion mobility in soils, reduced transpiration rate and impaired membrane transporter activity [21, 150]. To mitigate these effects, several mechanisms are activated, including ion homeostasis that maintain osmotic adjustment and turgor pressure. Rapid uptake and distribution of K is required for plant growth, regulated by a sophisticated network of potassium transporters [52]. In agreement with these observations, our results suggest that the K-fertilized eucalyptus, which displayed improved tree growth despite the water deficit, required increased K transport to sustain higher K needs [23]. By contrast, Na-fertilized eucalyptus with reduced tree growth compared to K-fertilized ones, displayed no increased activity of K transporters, especially those known to be involved in Na transport, such as NSCCs, HKTs, AKT and HAK [151].

With fertilization, regardless of the rainfall regime, K transport activity increased in K-deficient leaves compared to in K- and Na-fertilized ones, although to a lesser extent. Plants facing K-depletion can have increased transcription of K transport-related genes [152]. The majority of genes belong to NSCCs, channels that are permeable to a wide range of monovalent cations. In Arabidopsis guard cells, NSCCs are involved in stomatal regulation, and contribute to K loading into the xylem [153]. K-deficient eucalyptus leaves had increased AKT1, which mediates K+ uptake at low concentrations [154, 155], and HKT1, the high-affinity transporter also capable of mediating Na [156]. When we compared K transport in K- and Na-fertilized leaves, half the genes were both over and down-expressed. As observed with RR, no specific activity of transporters was revealed in Na-fertilized leaves, regardless of the rainfall regime. In all, 10 aquaporins (AQPs) were identified, with more PIPs specifically regulated according to RR, while NIPs and TIPs were correlated with the fertilization regime. AQPs play a crucial role in tolerance to drought stress. They are involved in the passive transport of water and small neutral solutes, and have been shown to be regulated in guard cells, thereby controlling stomatal closure [157–159]. AQPs are regulated at a transcriptional and post-translational level to avoid water loss [160]. Some AQPs may help maintain normal plant physiological processes, while others may help to adapt to or tolerate the stress condition [161]. PIPs and TIPs are more involved in water transport, while NIPs and SIPs are involved in solute transport [160]. With RR, sub-patterns of TIPs were revealed depending on the fertilization treatment, and all genes were globally down-regulated with RR, as already reported in the leaves of drought-stressed grapevine [162] and tobacco [163]. This could result in reduce membrane permeability and hence increase water conservation. Only one AQP was regulated with Na fertilization, a treatment that did not appear to trigger water stress. By contrast, fertilization affected more NIPs and TIPs with RR, but we were unable to identify a level of expression representative of each fertilization regime. In Arabidopsis roots and shoots, AQPs were found to be down-expressed under K deficiency [164, 165]. It is highly probable that interactions occur between AQPs and mineral fertilization, but no evidence for such interactions has been produced so far [166].

Overall, this study provides evidence that regulation of K transporters increased with K supply and decreased with K-deficiency in water-deficient eucalyptus. Conversely, K transporter regulation increased with K-deficiency regardless of the water regime. Moreover, rainfall reduction did not affect the activity of K transporters with low Na supply, including those known to be involved in Na transport. While different families of AQPs were regulated according to RR and the fertilization regime, their activity was mainly reduced under rainfall reduction. As both K and water transport are highly co-regulated, this analysis highlights the importance of designing multifactorial experiments to decipher plant responses to water deficiency.

## Conclusion

**Fig 7:**
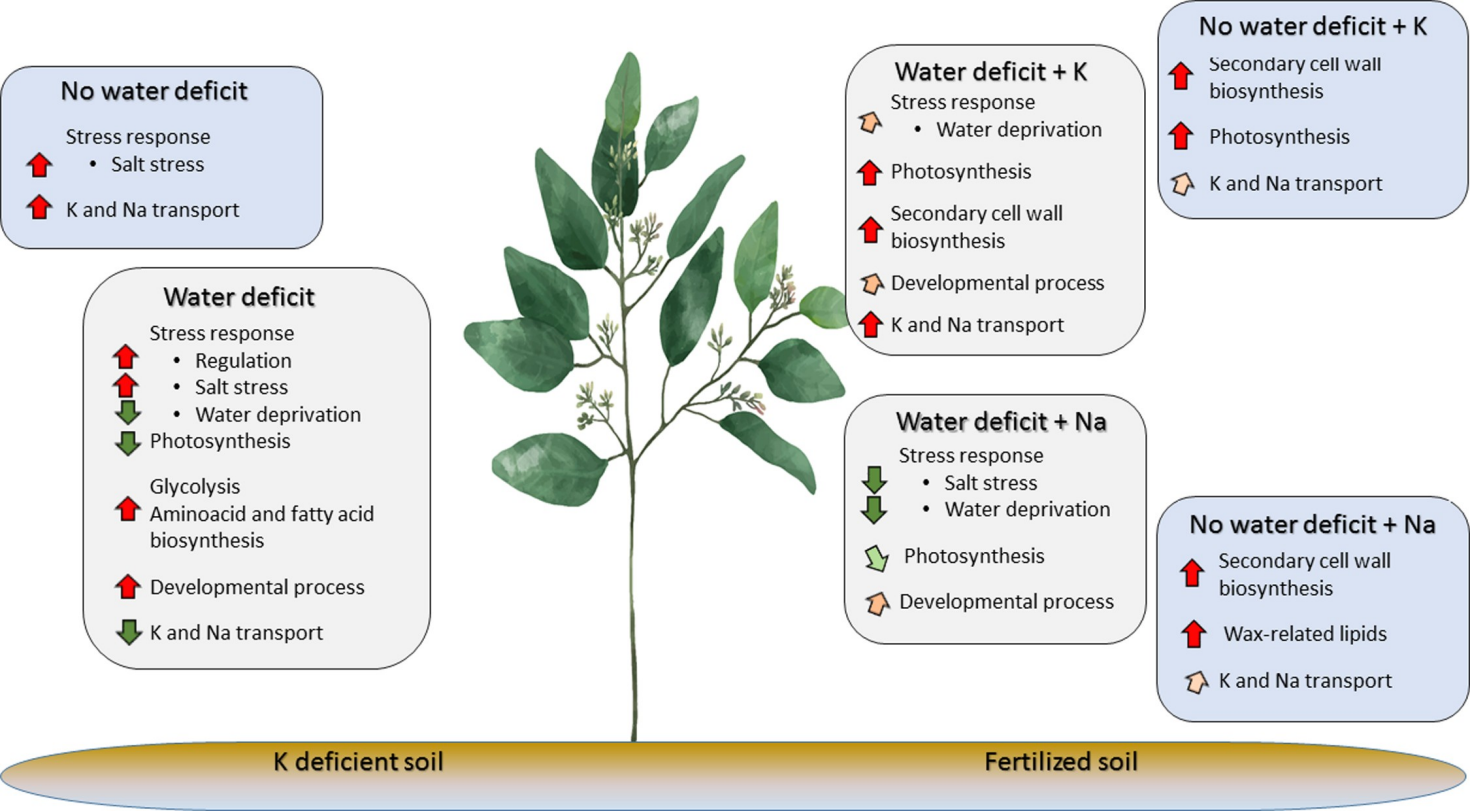
*Eucalyptus grandis* leaf response to water deficit and K and Na fertilization.

This is the first study to describe and annotate the leaf transcriptional response of water-deficient *Eucalyptus grandis* supplied with K or Na, and after two years of stress adaptation. This was made possible thanks to an experimental field trial that mimicked natural conditions in eucalyptus plantations in Brazil. Analysis of high-throughput data resulting from a multifactorial design was facilitated by recent advances in statistical methods, as well as co-expression network approaches. The measured transcriptional response was primarily influenced by rainfall reduction, unlike tree growth, although metabolic responses differed with the fertilization regime. Significant regulation of stress response was revealed, along with primary and secondary metabolism, secondary cell wall modification, photosynthesis, and specific patterns of cation transporters and aquaporins. A summary of the results is presented in Fig 7.

The most striking observations were 1) K-related water stress was detected, supporting field observations of an increase in tree water requirements with this treatment; 2) improved photosynthesis and increased cell wall regulation with K fertilization and water deficit, in agreement with the results of previous studies; 3) Na-fertilization mitigated the water and salt stress responses to water deficit and K deficiency and K fertilization, as well as the decrease in photosynthetic activity. While studying Na fertilization is significantly hampered by the physiological and genetic complexity of this trait, these results provide keys to decipher the intermediate response of Na-fertilized trees to water deficit observed in the field. This study demonstrated that higher-level understanding is needed to elucidate the biological mechanisms involved in Na functioning, as well as those involved in plant resistance to drought, in contrast to K fertilization. Identification of specific patterns of K and Na ions and water transporters provides opportunities for further investigation. K and Na ions and water transporters may be interesting targets for genetic improvement and clone selection with improved growth properties and drought resistance in K-deficient condition or partial Na fertilization. Overall, this study underlines the importance of using field experiments and multi-level approaches to study tree responses to abiotic stress involving complex biological processes.

## Supporting information

S1 Fig Mean temperature and rainfalls from 8 to 29 months after planting. (TIF)

S1 Table Counts of Multifactor DEGs and names of genes. (XLSX)

S2 Table List of genes in the Brown, Purple, Blue, GreenYellow, Lightcyan (functional analysis with enrichment in biological process, Kegg pathway and protein domain) networks, and correlation analysis of module-treatments (Correlation values and p-values). (XLSX)

S3 Table List of over-expressed and down-expressed C Rainfall, K Rainfall and Na Rainfall DEGs (log2 fold change and FDR; Functional analysis with enrichment in the biological process, Kegg pathway and protein domain). (XLSX)
