# Supplementary figures and images for: Distinct leaf transcriptomic response of water deficient Eucalyptus grandis submitted to potassium and sodium fertilization
Source: PLoS One. 2019 Jun 20;14(6):e0218528. doi: 10.1371/journal.pone.0218528 (PMC6586347; doi:10.1371/journal.pone.0218528)

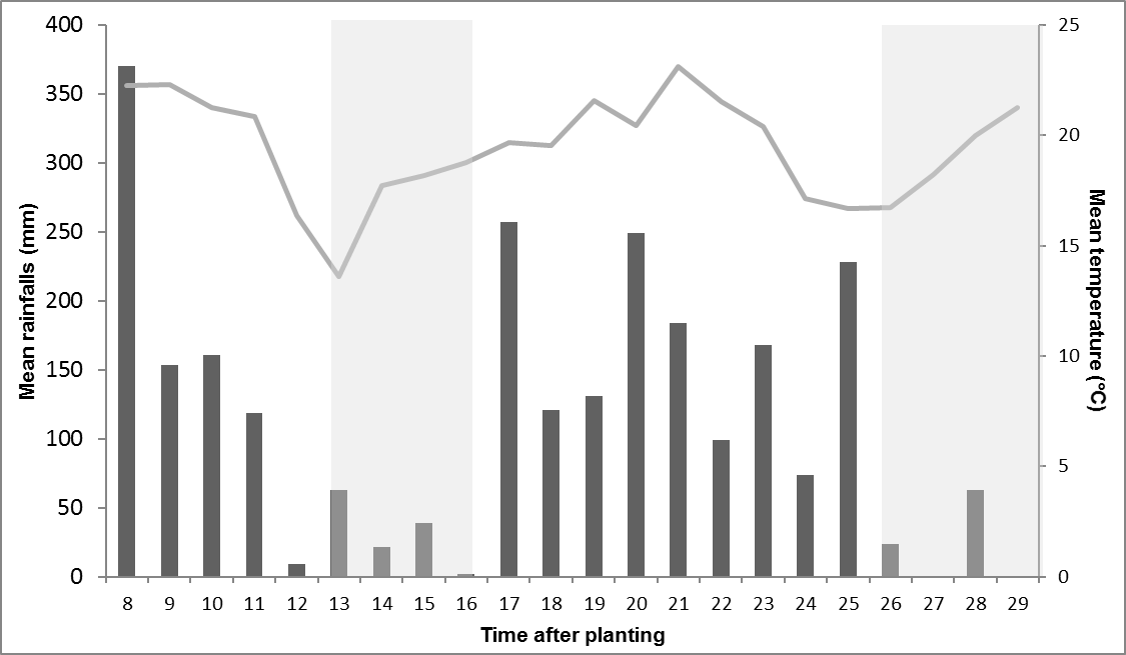

Supplement: S1 Fig — (TIF) [file pone.0218528.s001.tif]
